# Supplementary material for: Rapid and widespread white matter plasticity during an intensive reading intervention
Source: Nat Commun. 2018 Jun 8;9:2260. doi: 10.1038/s41467-018-04627-5 (PMC5993742; doi:10.1038/s41467-018-04627-5)
Supplement: Supplementary file 1 — Supplementary Information [file 41467_2018_4627_MOESM1_ESM.pdf]

Rapid and widespread white matter plasticity during an intensive reading intervention

Huber et al.

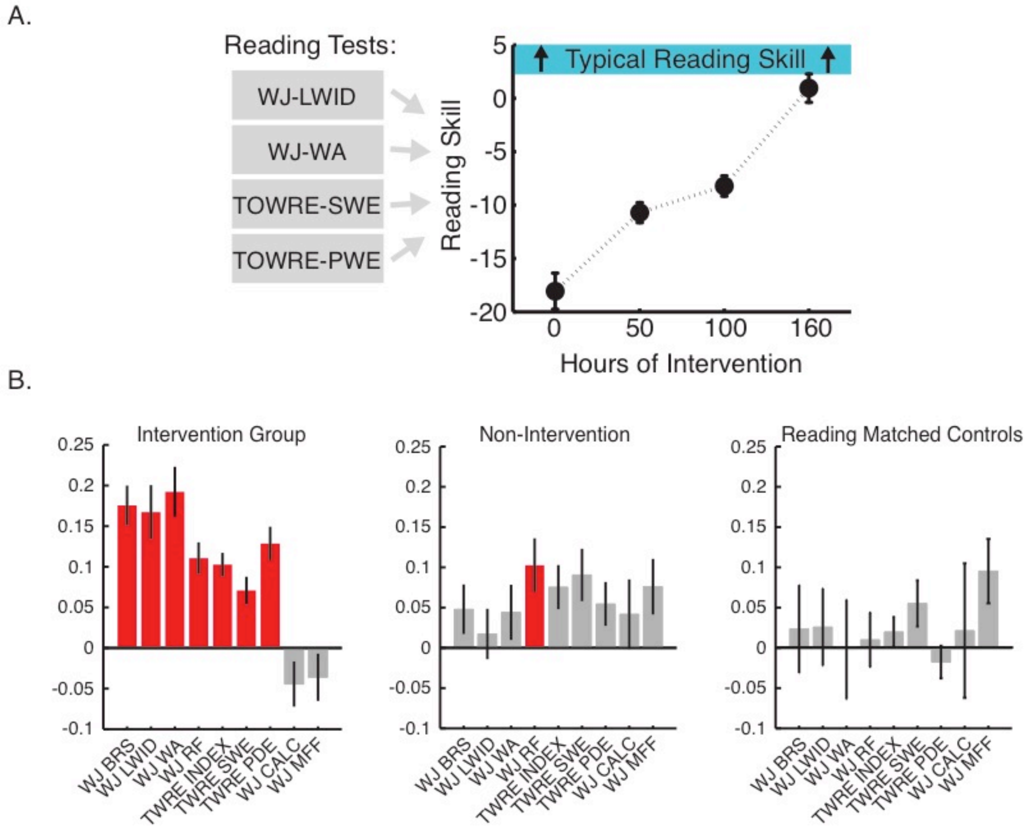

Supplementary figure 1. Intervention driven growth in reading performance. (A) We created a single summary index of reading skills based on conducting principal component analysis of the Woodcock Johnson and Test of Word Reading Efficiency standard scores (see Methods). Intervention driven change in this Reading Skill composite is plotted as a function of intervention hours and shows highly significant change (linear mixed effects model, fixed effect of intervention hours and random effect of subject, $p<10^{-9}$). (B) Linear growth in each of the standardized reading measures comprising the Reading Skill composite. In the intervention group, each of the reading subtests grew significantly during the intervention. For the full sample of non-intervention control subjects, we found a significant increase in performance in timed measures, reflecting practice with the tests. However, this practice-related improvement was only present for the children with good reading skills, and there was no change in any of the reading measures in the reading-skill matched control group.

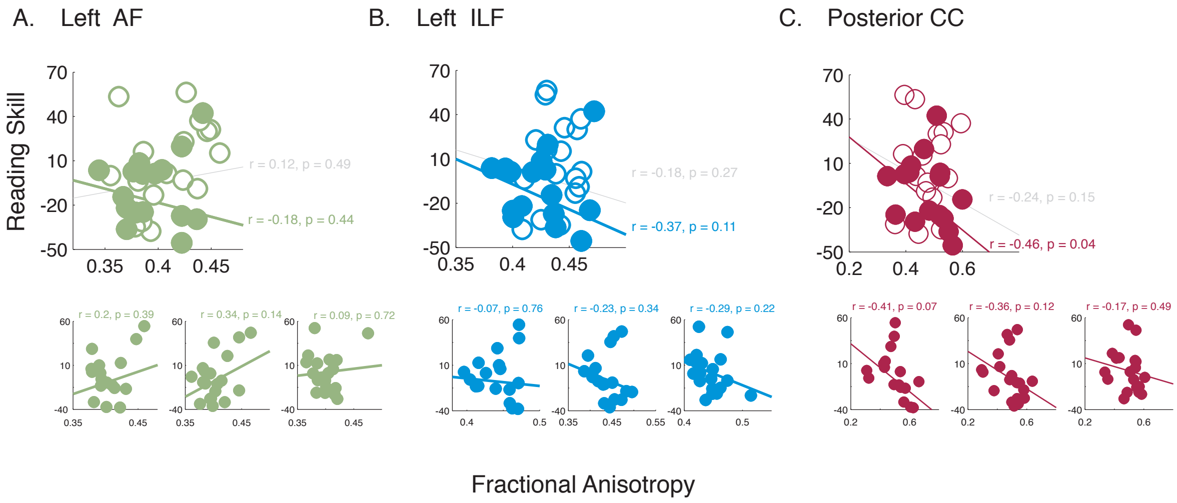

Supplementary figure 2. Tract properties vary as a function of pre-intervention reading skill. (A-C) Tract average FA is plotted as a function of pre-intervention (Session 1) reading skill. Best-fit lines plotted in gray give estimates for the full data set, while colored lines show fits for the intervention subjects, alone. Correlations for the intervention subjects are given in colored text below the value estimated for the full data set (in gray). Insets, below, show the cross-sectional correlation for Sessions 2-4 (left to right), during the intervention. Correlation values are reported for the intervention subjects, to highlight changes in the anatomy-behavior correlations that are specific to the intervention group.

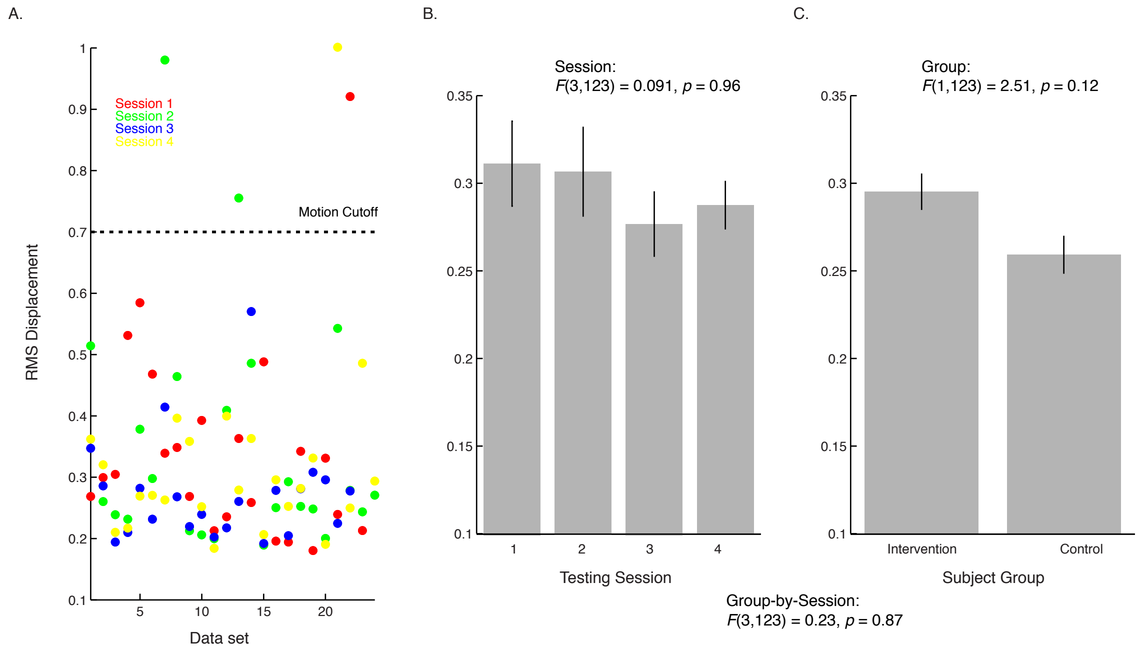

Supplementary figure 3. Analysis of subject motion. (A) Mean RMS displacement plotted for each intervention subject and session. Sessions are color coded (sessions 1-4 in red, blue, green, and yellow). Data sets included in subsequent analysis met the following criteria: (1) mean slice-by-slice RMS displacement < 0.7mm, (2) < 10% of volumes dropped or contained visible artifact. In total, we removed 9 of 93 total intervention datasets, and 3 of 52 control datasets. (B) Mean RMS motion is plotted for each session. Error bars represent standard error of the mean. Head motion did not differ across intervention sessions 1-4. (C) We found no group-by-session interaction in head motion, suggesting that changes in diffusivity within training, in the intervention group, could not be attributed to motion.

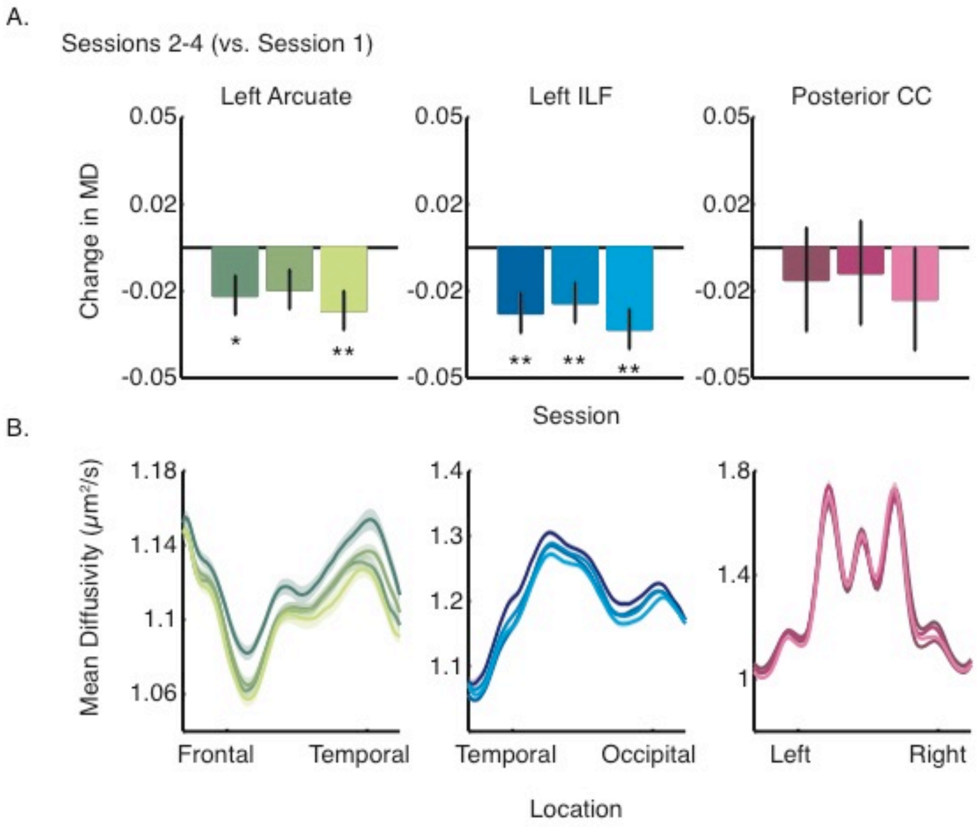

Supplementary figure 4. Changes in MD plotted on single session tractography for the intervention group. Intervention-driven changes do not depend on whether fiber tracts are identified independently for each session or in the multi-session concatenated data. (A-B) Values are selected based on tractography carried out on same-session data. Note that the regions of interest identified by the tractography algorithm will differ slightly between sessions. (A) Model predicted change in MD for each session (relative to baseline). Asterisks indicate significant decrease in MD for each session relative to the pre-intervention measurement (Session 1) at a Bonferroni corrected $p < 0.05$. (B) Tract profiles showing average mean diffusivity (MD) across subjects, measured at four time-points: pre-intervention (Session 1), after 2.5 weeks of intervention (Session 2), after 5 weeks of intervention (Session 3), and after 8 weeks of intervention (Session 4). Shaded error bars give ±1 standard error of the mean. Color values indicate session, ranging from darkest (Session 1) to brightest (Session 4) for each tract. Both the arcuate fasciculus (AF) and inferior longitudinal fasciculus (ILF) show a systematic decrease in MD over the course of intervention.

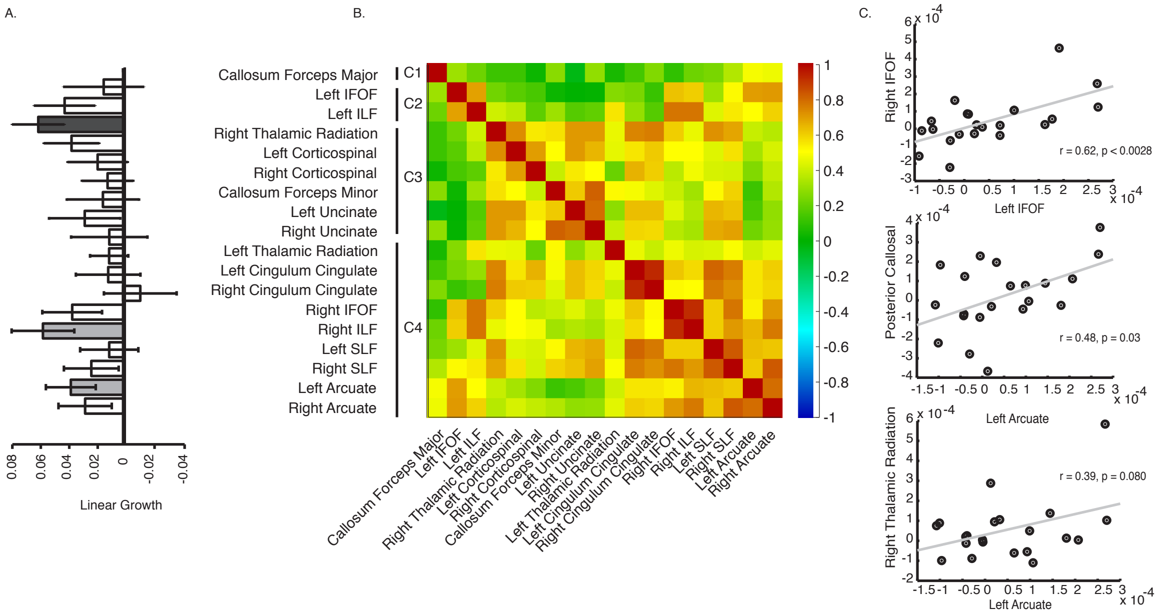

Supplementary figure 5. Reading intervention causes distributed changes in the white matter. (A) Change in FA as a function of intervention time (in hours) for 18 tracts. Tracts showing significant change (Bonferroni corrected $p < 0.05$) are indicated as dark gray filled bars. Tracts showing change at an uncorrected threshold ($p < 0.05$) are indicated as light gray filled bars. (B) Hierarchical clustering based on the correlations between linear growth rates. The heat map represents correlations between linear growth rates for pairs of tracts. The matrix is sorted according to hierarchical clustering of these correlations. (C) Scatter plots of individual growth rates for three pairs of tracts: left vs. right IFOF, AF vs. CC, and AF vs. right thalamic radiation.

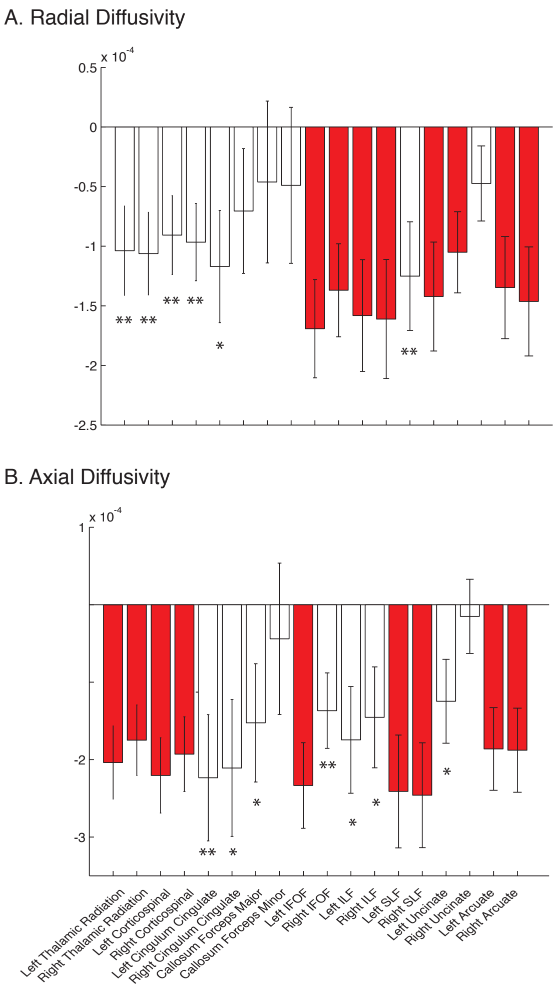

Supplementary figure 6. Change in additional diffusion as a function of intervention time (in hours) for individual tracts. Tracts showing significant change (Bonferroni corrected $p < 0.05$) are highlighted in red. Tracts with significant change before correction are indicated with a single (uncorrected $p < 0.05$) or double (uncorrected $p < 0.01$) asterisk.

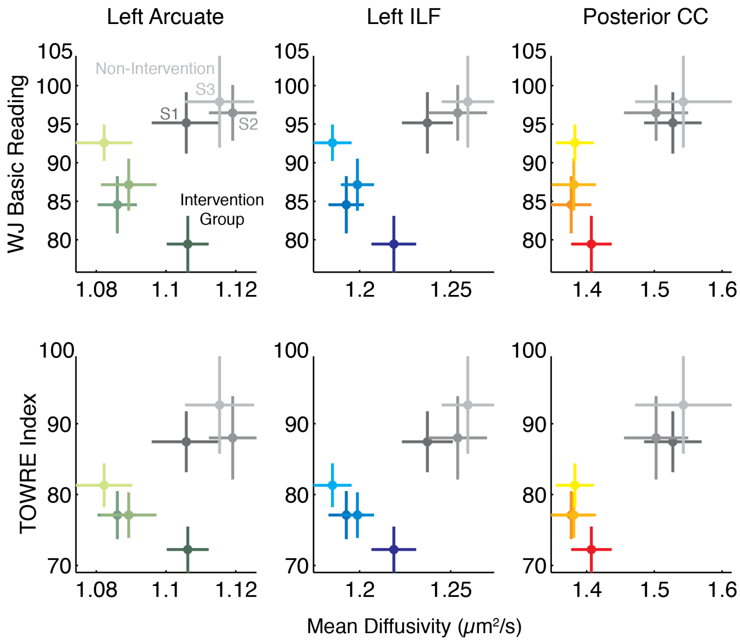

Supplementary figure 7. Reading scores and white matter tissue properties do not change systematically in the control group. Intervention data are plotted alongside the session-to-session means and standard errors for the full sample of control subjects. Because many of the control subjects completed fewer than 4 sessions, the control samples at each time point do not overlap perfectly. We omit session 4 due to the small number of available subjects. Session 1-3 data are shown in dark to lighter gray. Color-coding for the intervention subjects is the same as Figure 3, with brighter colors corresponding to later time points. Intervention subjects show large changes in behavior and white matter tissue properties between sessions 1, 2 and 3, but control subjects do not. Group by session interactions are reported in the main text of the Results.

TRACT	MD	FA
Left Thalamic Radiation	$r = 0.37$, $p = 0.021$	$r = -0.061$, $p = 0.72$
Right Thalamic Radiation	$r = 0.32$, $p = 0.049$	$r = -0.13$, $p = 0.44$
Left Corticospinal	$r = -0.042$, $p = 0.80$	$r = 0.35$, $p = 0.033$
Right Corticospinal	$r = 0.0041$, $p = 0.98$	$r = 0.22$, $p = 0.19$
Left Cingulum Cingulate	$r = 0.20$, $p = 0.24$	$r = 0.072$, $p = 0.67$
Right Cingulum Cingulate	$r = 0.15$, $p = 0.38$	$r = -0.11$, $p = 0.51$
Callosum Forceps Major	$r = 0.40$, $p = 0.013$	$r = -0.24$, $p = 0.047$
Callosum Forceps Minor	$r = 0.47$, $p = 0.0032$	$r = -0.32$, $p = 0.021$
Left IFOF	***r = 0.50, p = 0.0015***	$r = -0.002$, $p = 0.99$
Right IFOF	$r = 0.42$, $p = 0.0079$	$r = -0.14$, $p = 0.40$
Left ILF	***r = 0.51, p = 0.0010***	$r = -0.18$, $p = 0.27$
Right ILF	$r = 0.42$, $p = 0.0079$	$r = -0.34$, $p = 0.035$
Left SLF	$r = 0.23$, $p = 0.17$	$r = 0.10$, $p = 0.54$
Right SLF	$r = 0.16$, $p = 0.32$	$r = 0.082$, $p = 0.62$
Left Uncinate	$r = 0.24$, $p = 0.15$	$r = 0.21$, $p = 0.20$
Right Uncinate	$r = 0.37$, $p = 0.021$	$r = 0.091$, $p = 0.59$
Left Arcuate	$r = 0.28$, $p = 0.092$	$r = 0.12$, $p = 0.49$
Right Arcuate	$r = 0.15$, $p = 0.38$	$r = 0.18$, $p = 0.28$

Supplementary table 1. Tract properties and reading skills are correlated at baseline (pre-intervention). Correlations with pre-intervention Mean Diffusivity (MD) and Fractional Anisotropy (FA) are given for all tracts included in an exploratory analysis. Statistics were calculated using the full (Intervention and Control) sample of baseline (pre-intervention) Reading Skill scores. Tracts showing significant correlation at a conservative threshold (Bonferroni corrected $p < 0.05$) are highlighted in bold italic.

READING MEASURE	GROUP	TIME	INTERACTION
Reading Skill (PCA)	$F(1,124) = 8.55$, $p = 0.0041$	$F(1,124) = 5.34$, $p = 0.022$	$F(1,124) = 9.38$, $p = 0.0027$
WJ-BRS	$F(1,124) = 9.59$, $p = 0.0024$	$F(1,124) = 1.86$, $p = 0.17$	$F(1,124) = 8.64$, $p = 0.0039$
TOWRE Index	$F(1,124) = 6.52$, $p = 0.012$	$F(1,124) = 9.28$, $p = 0.0028$	$F(1,124) = 0.85$, $p = 0.36$
WJ-CALC	$F(1,98) = 8.79$, $p = 0.0038$	$F(1,98) = 0.92$, $p = 0.34$	$F(1,98) = 2.78$, $p = 0.099$
WJ-BRS	$F(1,94) = 0.16$, $p = 0.68$	$F(1,94) = 0.19$, $p = 0.67$	$F(1,94) = 4.22$, $p = 0.042$
TOWRE Index	$F(1,94) = 1.12$, $p = 0.29$	$F(1,94) = 0.24$, $p = 0.63$	$F(1,94) = 4.069$, $p = 0.047$
WJ-CALC	$F(1,94) = 4.10$, $p = 0.046$	$F(1,94) = 0.31$, $p = 0.58$	$F(1,94) = 1.13$, $p = 0.29$

Supplementary table 2. Behavioral effects. Growth in reading skill was specific to the intervention group. Results for the full control sample (n = 19), including 10 skilled reading control subjects, are shown at the top. In this group, performance improved with repeated testing for the timed measures (TOWRE and Reading Fluency). In all control subjects, untimed measures (WJ Basic Reading) were stable, showing no change over 8 weeks. Results for a subset of reading-matched controls (n = 9) are shown below, shaded in gray.

	DAYS	GROUP	DAYS*GROUP	DAYS^2	DAYS^2*GROUP
**AF MD**	$F(1,123) = 0.90$, $p = 0.35$	$F(1,123) =.62$, $p = 0.0067$	$F(1,123) = 6.1$, $p = 0.015$	$F(1,123) = 0.056$, $p = 0.81$	$F(1,123) = 0.92$, $p = 0.34$
**ILF MD**	$F(1,123) = 4.15$, $p = 0.044$	$F(1,123) = 8.56$, $p = 0.0041$	$F(1,123) = 11.28$, $p = 0.0010$	$F(1,123) = 0.42$, $p = 0.52$	$F(1,123) = 0.00022$, $p = 0.99$
**CC MD**	$F(1,123) = 0.42$, $p =0.519$	$F(1,123) = 6.41$, $p = 0.013$	$F(1,123) = 0.31$, $p = 0.58$	$F(1,123) = 2.21$, $p = 0.14$	$F(1,123) = 0.11$, $p = 0.74$
**AF FA**	$F(1,123) = 0.14$, $p = 0.71$	$F(1,123) = 0.0064$, $p = 0.94$	$F(1,123) = 1.41$, $p = 0.24$	$F(1,123) = 0.29$, $p = 0.59$	$F(1,123) = 3.81$, $p = 0.053$
**ILF FA**	$F(1,123) = 2.84$, $p = 0.094$	$F(1,123) = 0.48$, $p = 0.49$	$F(1,123) = 0.25$, $p = 0.62$	$F(1,123) = 1.64$, $p = 0.20$	$F(1,123) = 3.76$, $p = 0.055$
**CC FA**	$F(1,123) = 0.65$, $p = 0.42$	$F(1,123) = 0.32$, $p = 0.57$	$F(1,123) = 0.002$, $p = 0.96$	$F(1,123) = 0.33$, $p = 0.56$	$F(1,123) = 0.18$, $p = 0.67$

Supplementary table 3. Linear and quadratic effects in the white matter. Results for a linear mixed effects model predicting white matter properties (mean diffusivity, MD, and fractional anisotropy, FA) for the left arcuate (AF), inferior longitudinal fasciculus (ILF) and posterior callosal connections (CC) as a function of intervention time (in days) and subject group (intervention vs. non-intervention control),

	READING SKILL	POSTERIOR CC	LEFT ILF	LEFT ARCUATE
**Session 1 vs. 2**	$F(19,19) = 1.088$, $p > 0.05$	$F(19,19) = 1.17$, $p > 0.05$	$F(19,19) = 1.045$, $p > 0.05$	$F(19,19) = 1.16$, $p > 0.05$
**Session 1 vs. 3**	$F(19,19) = 1.24$, $p > 0.05$	$F(19,19) = 1.30$, $p > 0.05$	$F(19,19) = 1.25$, $p > 0.05$	$F(19,19) = 1.075$, $p > 0.05$
**Session 1 vs. 4**	$F(19,19) = 1.49$, $p > 0.05$	$F(19,19) = 1.70$, $p > 0.05$	$F(19,19) = 1.98$, $p > 0.05$	$F(19,19) = 2.27$, $p > 0.01$

Supplementary table 4. Variance in white matter and reading skills is well matched across sessions. F-statistics in each cell represent the ratio of variance across time points for each white matter tract, and the Reading Skill composite, calculated with the larger of the 2 variances in the numerator. Statistics were computed using the 20 intervention subjects with the full set of 4 MRI data points.

**ILF VS. READING SKILL**	**AF VS. READING SKILL**	**ILF VS. AF**
$F(1,68) = 4.46$, $p = 0.038$	$F(1,68) = 3.98$, $p = 0.050$	$F(1,69) = 335.42$, $p > 10^{-27}$

Supplementary table 5. Baseline normalized analysis of learning trajectories. Results of a mixed effects model predicting mean diffusivity, relative to baseline, in the left inferior longitudinal fasciculus (ILF), and the left arcuate (AF) from reading skill throughout the intervention, and mean diffusivity, relative to baseline, in the left inferior longitudinal fasciculus (ILF) as a function of mean diffusivity, relative to baseline, in the left arcuate (AF).

TRACT	MEAN DIFFUSIVITY	FRACTIONAL ANISOTROPY
Left Thalamic Radiation	*r* = -0.035, *p* = 0.81	*r* = 0.0016, *p* =0.99
Right Thalamic Radiation	*r* = 0.079, *p* = 0.58	*r* = -0.17, *p* = 0.22
Left Corticospinal	*r* = -0.052, *p* = 0.72	*r* = 0.017, *p* = 0.91
Right Corticospinal	*r* = 0.062, *p* = 0.67	*r* = -0.025, *p* = 0.86
Left Cingulum Cingulate	*r* = -0.042, *p* = 0.77	*r* = 0.011, *p* = 0.94
Right Cingulum Cingulate	*r* = 0.13, *p* = 0.36	*r* = -0.027, *p* = 0.85
Callosum Forceps Major	*r* = -0.15, *p* = 0.29	*r* = 0.15, *p* = 0.31
Callosum Forceps Minor	*r* = 0.030, *p* = 0.84	*r* = -0.049, *p* = 0.73
Left IFOF	*r* = -0.012, *p* = 0.93	*r* = 0.081, *p* = 0.081
Right IFOF	*r* = 0.17, *p* = 0.23	*r* = -0.023, *p* = 0.87
Left ILF	*r* = 0.070, *p* = 0.62	*r* = 0.021, *p* = 0.88
Right ILF	*r* = 0.0013, *p* = 0.99	*r* = -0.057, *p* = 0.69
Left SLF	*r* = -0.080, *p* = 0.58	*r* = -0.045, *p* = 0.76
Right SLF	*r* = 0.053, *p* = 0.71	*r* = -0.048, *p* = 0.74
Left Uncinate	*r* = -0.019, *p* = 0.90	*r* = 0.0050, *p* = 0.97
Right Uncinate	*r* = 0.099, *p* = 0.49	*r* = -0.16, *p* = 0.25
Left Arcuate	*r* = -0.046, *p* = 0.75	*r* = -0.059, *p* = 0.68
Right Arcuate	*r* = 0.059, *p* = 0.68	*r* = -0.13, *p* = 0.38

Supplementary table 6. Non-Intervention MD and FA Versus Reading Skill Over Time. Cells show p-values based on a mixed linear model predicting session-to-session changes in Reading Skill composite scores from in mean diffusivity (MD) and fractional anisotropy (FA) in the non-intervention Control subjects. Pearson correlations between mean-centered MD/FA and mean-centered reading score are provided as an index of effect size. Consistent with the stability of both reading scores and diffusion properties in the control group, no tracts change in relation to Reading Skill in the Control group.

	WJ-BRS SS	TOWRE-Index	WJ-LW Raw	WJ-WA Raw	TOWRE-SWE Raw	TOWRE-PDE Raw
**ILF MD**	r = 0.11, p = 0.63	r = -0.27, p = 0.14	r = -0.11, p = 0.61	r = -0.30, p = 0.17	r = -0.43, p = 0.039	r = -0.19, p = 0.39
**AF MD**	r = 0.098, p = 0.66	r = -0.24, p = 0.20	r = 0.029, p = 0.90	r = -0.30, p = 0.16	r = -0.47, p = 0.023	r = -0.21, p = 0.33
**ILF FA**	r = 0.048, p = 0.80	r = 0.36, p = 0.051	r = 0.040, p = 0.86	r = 0.059, p = 0.79	r = -0.034, p = 0.88	r = 0.16, p = 0.47
**AF FA**	r = -0.03, p = 0.87	r = 0.42, p = 0.02	r = 0.066, p = 0.77	r = 0.085, p = 0.70	r = 0.30, p = 0.16	r = 0.18, p = 0.41

Supplementary table 7. Session 2 vs. Session 1 Difference Scores. Each cell gives the Session 2 vs. Session 1 difference score for the specified tract (left arcuate, AF, or left ILF) and parameter (mean diffusivity, MD, or fractional anisotropy, FA) and reading measure (Woodcock-Johnson or TOWRE subtests, from left to right: WJ Basic Reading Standard Score, TOWRE Index, WJ Letter-Word ID, WJ Word Attack, TOWRE Sight Word Efficiency, TOWRE Phonemic Decoding Efficiency).

TRACT (FA)	READING SKILL	BASIC READING	TOWRE INDEX	FLUENCY
Left Thalamic Radiation	$r = 0.020$, $p = 0.86$	$r = -0.061$, $p = 0.59$	$r = 0.095$, $p = 0.40$	$r = 0.031$, $p = 0.78$
Right Thalamic Radiation	$r = 0.13$, $p = 0.25$	$r = 0.082$, $p = 0.46$	$r = 0.12$, $p = 0.30$	$r = 0.30$, $p = 0.0076$
Left Corticospinal	$r = 0.14$, $p = 0.20$	$r = 0.067$, $p = 0.55$	$r = 0.15$, $p = 0.19$	$r = 0.21$, $p = 0.065$
Right Corticospinal	$r = 0.060$, $p = 0.60$	$r = 0.055$, $p = 0.62$	$r = -0.0020$, $p = 0.99$	$r = 0.16$, $p = 0.14$
Left Cingulum Cingulate	$r = 0.045$, $p = 0.69$	$r = 0.029$, $p = 0.80$	$r = -0.0060$, $p = 0.96$	$r = 0.044$, $p = 0.70$
Right Cingulum Cingulate	$r = -0.093$, $p = 0.41$	$r = -0.14$, $p = 0.23$	$r = -0.036$, $p = 0.75$	$r = -0.0074$, $p = 0.95$
Callosum Forceps Major	$r = 0.014$, $p = 0.90$	$r = -0.055$, $p = 0.62$	$r = 0.15$, $p = 0.19$	$r = 0.12$, $p = 0.30$
Callosum Forceps Minor	$r = -0.038$, $p = 0.74$	$r = -0.058$, $p = 0.61$	$r = 0.047$, $p = 0.68$	$r = 0.063$, $p = 0.58$
Left IFOF	$r = 0.10$, $p = 0.36$	$r = 0.098$, $p = 0.39$	$r = 0.11$, $p = 0.31$	$r = 0.14$, $p = 0.23$
Right IFOF	$r = 0.13$, $p=0.24$	$r = 0.13$, $p = 0.24$	$r = 0.14$, $p = 0.20$	$r = 0.26$, $p = 0.021$
Left ILF	$r = 0.20$, $p=0.08$	$r = 0.13$, $p = 0.25$	$r = 0.26$, $p = 0.019$	$r = 0.19$, $p = 0.093$
Right ILF	$r = 0.17$, $p=0.14$	$r = 0.14$, $p = 0.23$	$r = 0.18$, $p = 0.10$	$r = 0.26$, $p = 0.020$
Left SLF	$r = 0.032$, $p=0.77$	$r = -0.039$, $p = 0.73$	$r = 0.062$, $p = 0.58$	$r = 0.051$, $p = 0.66$
Right SLF	$r = 0.10$, $p=0.36$	$r = 0.088$, $p = 0.44$	$r = 0.063$, $p = 0.58$	$r = 0.14$, $p = 0.21$
Left Uncinate	$r = 0.15$, $p=0.18$	$r = 0.17$, $p = 0.12$	$r = 0.028$, $p = 0.80$	$r = 0.29$, $p = 0.0096$
Right Uncinate	$r = -0.063$, $p=0.58$	$r = -0.020$, $p = 0.86$	$r = -0.17$, $p = 0.12$	$r = -0.021$, $p = 0.86$
Left Arcuate	$r = 0.078$, $p=0.49$	$r = -0.016$, $p = 0.89$	$r = 0.17$, $p = 0.12$	$r = 0.078$, $p = 0.49$
Right Arcuate	$r = 0.11$, $p=0.32$	$r = 0.040$, $p = 0.72$	$r = 0.19$, $p = 0.096$	$r = 0.11$, $p = 0.33$

Supplementary table 8. FA Change Versus Reading Skill Change during the intervention. Cells show p-values based on a mixed linear model predicting session-to-session changes Reading Skill composite score, Woodcock-Johnson Basic Reading scores, TOWRE index scores, and Woodcock-Johnson Reading Fluency from changes in fractional anisotropy (FA) at each time point during the intervention. Pearson correlations between mean-centered FA and mean-centered reading score are provided as an index of effect size.

ILF MD	AF MD	ILF FA	AF FA
$r = 0.11$, $p = 0.65$	$r = -0.066$, $p = 0.78$	$r = -0.19$, $p = 0.41$	$r = -0.16$, $p = 0.49$

Supplementary table 9. Individual rates of change in diffusion measures vs. Reading Skill. Each subject's rate of change for the Reading Skill composite, and for each tract (left arcuate, AF, or left ILF) and parameter (mean diffusivity, MD, or fractional anisotropy, FA), is estimated from the linear fit to intervention hours. All subjects' rates of change in reading and white matter properties are then used to estimate the correlation between the magnitude of change in reading and white matter.

TRACT	MEAN DIFFUSIVITY	FRACTIONAL ANISOTROPY
Left Thalamic Radiation	$F(1,67) = 6.38, p = 0.014$	$F(1,67) = 0.44, p = 0.51$
Right Thalamic Radiation	***F(1,67) = 11.70, p = 0.0011***	$F(1,67) = 1.41, p = 0.24$
Left Corticospinal	***F(1,67) = 16.69, p = 0.00012***	$F(1,67) = 3.13, p = 0.081$
Right Corticospinal	$F(1,67) = 8.74, p = 0.0043$	$F(1,67) = 0.77, p = 0.38$
Left Cingulum Cingulate	$F(1,67) = 7.26, p = 0.0089$	$F(1,67) = 1.05, p = 0.31$
Right Cingulum Cingulate	$F(1,67) = 4.30, p = 0.042$	$F(1,67) = 0.28, p = 0.60$
Callosum Forceps Major	$F(1,67) = 0.62, p = 0.44$	$F(1,67) = 2.38, p = 0.13$
Callosum Forceps Minor	$F(1,67) = 3.56, p = 0.063$	$F(1,67) = 1.96, p = 0.17$
Left IFOF	***F(1,67) = 12.29, p = 0.00082***	$F(1,67) = 7.02, p = 0.010$
Right IFOF	***F(1,67) = 21.28, p < 10^{-4}***	$F(1,67) = 2.96, p = 0.090$
Left ILF	$F(1,67) = 7.75, p = 0.0070$	$F(1,67) = 6.45, p = 6.45$
Right ILF	***F(1,67) = 9.68, p = 0.0027***	$F(1,67) = 4.38, p = 0.040$
Left SLF	$F(1,67) = 6.56, p = 0.013$	$F(1,67) = 0.11, p = 0.74$
Right SLF	***F(1,67) = 9.69, p = 0.0027***	$F(1,67) = 0.0048, p = 0.95$
Left Uncinate	$F(1,67) = 4.88, p = 0.031$	$F(1,67) = 4.29, p = 0.042$
Right Uncinate	$F(1,67) = 3.92, p = 0.052$	$F(1,67) = 1.69, p = 0.20$
Left Arcuate	$F(1,67) = 6.91, p = 0.011$	$F(1,67) = 2.85, p = 0.096$
Right Arcuate	$F(1,67) = 8.95, p = 0.0039$	***F(1,67) = 12.27, p = 0.00083***

Supplementary table 10. Exploratory Analysis of Intervention-Driven Change. Intervention-driven changes in the white matter are quantified based on the interaction effect (Group-by-Session; linear-mixed effects model with fixed effects of Group (Intervention vs. Control) and Session (1-2), and random effects of subject) in a large collection of white matter tracts. Intervention driven change in MD and FA is reported for each tract. For this exploratory analysis we use a conservative Bonferroni correction, and tracts showing significant change in the Intervention group (Bonferroni corrected $p < 0.05$) are highlighted in bold italic.

TRACT	MD RELIABILITY	FA RELIABILITY
Left Thalamic Radiation	$r = 0.77$	$r = 0.72$
Right Thalamic Radiation	$r = 0.72$	$r = 0.65$
Left Corticospinal	$r = 0.67$	$r = 0.61$
Right Corticospinal	$r = 0.88$	$r = 0.68$
Left Cingulum Cingulate	$r = 0.30$	$r = 0.76$
Right Cingulum Cingulate	$r = 0.60$	$r = 0.75$
Callosum Forceps Major	$r = 0.96$	$r = 0.96$
Callosum Forceps Minor	$r = 0.83$	$r = 0.90$
Left IFOF	$r = 0.75$	$r = 0.71$
Right IFOF	$r = 0.92$	$r = 0.74$
Left ILF	$r = 0.82$	$r = 0.59$
Right ILF	$r = 0.90$	$r = 0.58$
Left SLF	$r = 0.39$	$r = 0.66$
Right SLF	$r = 0.66$	$r = 0.84$
Left Uncinate	$r = 0.07$	$r = 0.73$
Right Uncinate	$r = 0.72$	$r = 0.87$
Left Arcuate	$r = 0.22$	$r = 0.88$
Right Arcuate	$r = 0.45$	$r = 0.88$

Supplementary table 11. Test re-test reliability, quantified using Pearson's r, for the full set of 18 tracts for the control subjects with at least 2 sessions of data ($n = 14$). In control subjects, the median reliability across tracts was $r = 0.73$ for mean diffusivity (MD) and $r = 0.76$ for fractional anisotropy (FA). For MD, reliability was reduced in a few tracts by the presence of outliers. Removing these outliers increased estimated reliability (e.g., $r = 0.59$ in the left arcuate), resulting in a median reliability of 0.78 for MD after outlier correction.
